# Laser Treatment of Peri-Implantitis: A Systematic Review of Radiographic Outcomes

**DOI:** 10.3390/dj10020020

**Published:** 2022-01-28

**Authors:** Miriam Ting, Leela Subhashini C. Alluri, John G. Sulewski, Jon B. Suzuki, Andre Paes Batista da Silva

**Affiliations:** 1 Think Dental Learning Institute, Paoli, PA 19301, USA; 2 Department of Periodontics, Meharry Medical College School of Dentistry, Nashville, TN 37208, USA; lalluri@mmc.edu; 3 The Institute for Advanced Dental Technologies, Huntington Woods, MI 48070, USA; jsulewski09@gmail.com; 4 University of Maryland, Baltimore, MD 21201, USA; jon.suzuki@temple.edu; 5 University of Washington, Seattle, WA 98015, USA; 6 Nova Southeastern University, Ft. Lauderdale, FL 33314, USA; 7 Department of Periodontics, Case Western Reserve University School of Dental Medicine, Cleveland, OH 44106, USA; axp460@case.edu

**Keywords:** systematic review, peri-implant disease, peri-implantitis, laser, radiographic, radiograph

## Abstract

(1) Background: This systematic review aimed to evaluate the effects of laser therapy on radiographic bone level (RBL) changes in peri-implantitis defects. (2) Methods: A literature search with defined inclusion criteria was performed. PubMed, Web of Science, Cochrane Library, and Google Scholar were searched through September 2020. The evaluated primary outcomes were RBL changes. In studies that reported RBL data, corresponding secondary clinical outcomes were probing depth (PD), bleeding on probing (BOP), and clinical attachment level (CAL). (3) Results: Thirteen articles were selected for data extraction and risk of bias assessment. Eight studies showed evidence of RBL gain in the laser groups compared to baseline, but did not report the statistical significance. Eight of these 13 studies reported comparisons to control. Five of the eight studies did not show RBL gain in the laser groups compared to control. In the laser groups compared to baseline, 11 of 13 reported reduced PD, and 6 of 13 reported significantly reduced BOP. Compared to the control, eight of the eight reported reduction of PD, and three of six reported significantly reduced BOP. Statistical significance was not consistently reported. (4) Conclusions: Within the limits of this systematic review, laser treatment may promote bone gain in peri-implantitis defects, may reduce BOP and PDs, and may be comparable to mechanical therapy. However, definitive conclusions can only be made with statistically significant data, which were found lacking in the currently available studies. This systematic review was registered with the National Institute for Health Research, international prospective register of systematic reviews (PROSPERO): CRD42020207972.

## 1. Introduction

The increasing usage of implants to rehabilitate the edentulous alveolar ridge has led to the higher frequency of peri-implant diseases, classified as peri-implant mucositis or peri-implantitis [1,2]. Peri-implant mucositis is a reversible inflammatory lesion that occurs in the soft tissues surrounding the endosseous dental implants [3]. Untreated peri-implant mucositis develops a radiographic progressive bone loss around the osseointegrated implant, resulting in peri-implantitis [4,5]. The progression of peri-implantitis is non-linear and accelerating; it manifests as a circumferential pattern of bone loss apical to the implant platform [5]. The weighted mean prevalence of peri-implantitis has been estimated at 22% [6]. The primary etiology of peri-implant diseases is microbial biofilm [5]. An increased risk of peri-implantitis is reported in patients with a previous history of chronic periodontitis, poor periodontal maintenance compliance, and inadequate plaque control [5,7].

No single peri-implantitis treatment protocol is recognized, despite the availability of several treatment options. Treatment alternatives include non-surgical therapy with and without adjunctive use of local delivery antibiotics, lasers, and surgical therapy [7]. Non-surgical therapy consists of mechanical debridement (MD) of implant surfaces [8]. However, conventional mechanical therapy leads to increased roughness of the implant surface and oral pathogen retention. Mechanical therapy with adjunctive use of local antibiotics can reduce bleeding on probing (BOP) and probing depth (PD) [9]. The goal of surgical therapy is to create access for the debridement and decontamination of the implant surface [10]. Guided bone regeneration techniques have been used to enhance bone fill in peri-implant defects [11].

Laser therapy is bactericidal, does not alter the implant surface morphology when used properly, and can induce new bone formation [12]. Various laser systems, such as diode, neodymium: yttrium-aluminum-garnet (Nd:YAG), erbium: yttrium-aluminum-garnet (Er:YAG), and carbon dioxide (CO_2_), have been used for the treatment of peri-implantitis [13]. CO_2_ and diode lasers have been used for the decontamination of the implant surface [14,15]. Nd:YAG and Er:YAG lasers at low-intensity have bactericidal effects [16,17]. Er:YAG lasers have been utilized in both surgical and non-surgical therapy [18,19,20,21,22]. Therefore, when used to decontaminate and regenerate peri-implant bone defects, dental lasers may be a viable option for positively affecting RBL changes during peri-implantitis treatment. The aim of this review is to systematically evaluate the effect of high-intensity laser therapy on peri-implantitis defects by assessing the bone changes using radiographic methods.

## 2. Materials and Methods

### 2.1. Focus Question

What is the radiographic osseous response in peri-implant defects after laser-assisted peri-implantitis treatment? The following were addressed in this focus question (PICOS): Participants: humans diagnosed with peri-implantitis; Interventions: laser-assisted peri-implantitis therapy; Comparisons: treated sites vs. control/baseline; Outcomes: (1) primary: RBL changes, (2) secondary: CAL, BOP, PD; and Study design: descriptive studies. High-intensity laser usage that results in ablation and removal of gingival crevicular epithelium is categorized as a surgical treatment.

### 2.2. Literature Search and Study Design

**Figure 1:**
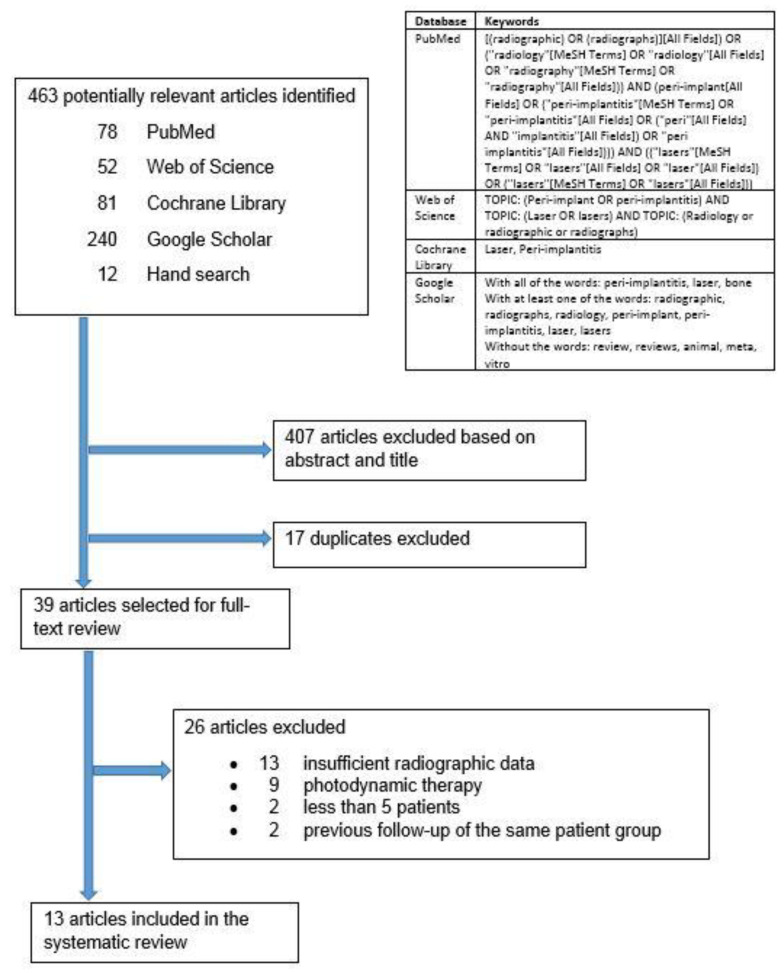
Search strategy.

The electronic databases PubMed, Web of Science, Cochrane Library, and Google Scholar were searched up to September 2020 (Figure 1). Google Scholar was also searched for gray literature. Additional hand searching of laser-related research was performed on the reference list of the selected articles. Experts in the field of dental laser-related research were consulted for additional articles. Corresponding authors of the selected articles were contacted to request any additional radiographic data or information regarding their studies and to suggest relevant new articles. Corresponding authors who responded did not provide any additional data. This systematic review was registered with the National Institute for Health Research, international prospective register of systematic reviews (PROSPERO): CRD42020207972. There were no amendments to the submitted protocol. This systematic review was performed in accordance with the PRISMA (Preferred Reporting Items for Systematic Reviews and Meta-Analyses) guidelines.

### 2.3. Inclusion Criteria

- Patients diagnosed with peri-implantitis, reported as inflamed peri-implant pockets 4 mm or more in depth and/or loss of supporting peri-implant bone, were included.
- Clinical studies with high-intensity laser therapy of peri-implant defects were included. High intensity laser usage that results in ablation and removal of gingival crevicular epithelium were categorized as surgical treatments.
- Studies with sufficient radiographic data for at least five patients were included.
- Clinical trials reporting radiographic effects of laser treatment on human peri-implant diseased periodontium were included.
- Non-English articles were included, but were selected for full-text analysis only if an English translation were available.

### 2.4. Exclusion Criteria

- All in vitro, cadaver, and animal studies were excluded.
- Photodynamic therapy studies were excluded.
- Non-surgical studies with low-intensity laser therapy that do not result in ablation or removal of gingival epithelium were excluded.
- Conference abstracts and posters were excluded.

### 2.5. Screening, Selection, and Data Extraction

Three reviewers (LSA, JGS, and MT) independently screened the “Title and Abstract”. Articles that did not meet the inclusion criteria were excluded. Articles were included for full-text screening if there were any doubt. The full text was then independently analyzed by the three reviewers (LSA, JGS, and MT). Data extraction of final selected articles was also independently performed by the same three reviewers with a previous pilot-tested data extraction sheet. The independently extracted data were cross-referenced among reviewers for accuracy and completeness. All disagreements pertaining to the literature screening, selection, and data extraction were resolved by discussion with a fourth reviewer (JBS). The evaluated primary outcome was RBL changes, and only studies that reported this were included. The corresponding secondary clinical outcomes PD, BOP, and CAL were also reported for these included studies.

### 2.6. Risk of Bias Assessment

The risk of bias (Table 1) was assessed using the risk of bias tool by the Office of Health Assessment and Translation (OHAT) [23]. The same three reviewers (LSA, JGS, and MT) independently scored the risk of bias, and disagreements were resolved through discussion with a fourth reviewer (AP).

## 3. Results

### 3.1. Search Results

The search yielded 463 reviews: 78 in PubMed, 52 in Web of Science, 81 in Cochrane Library, 240 in Google Scholar, and 12 from hand search (Figure 1). After the title and abstract screening, the duplicates were removed, and 39 articles remained for full-text analysis. After full-text analysis, 26 were eliminated: 13 for having insufficient radiographic data [18,21,34,35,36,37,38,39,40,41,42,43,44], nine for focusing on photodynamic therapy [45,46,47,48,49,50,51,52,53], two for less than five patients [54,55], and two for being previous follow up publications of the same patient group [20,56]. Only 13 articles remained for data extraction (Table 2, Table 3, Table 4, Table 5, Table 6, Table 7 and Table 8).

### 3.2. Quality of Evidence

The risk of bias (Table 1) of the selected six randomized trials [19,22,24,25,32,33] were mostly “definitely or probably low risk of bias”, and the risk of bias for the other seven non-randomized studies [14,26,27,28,29,30,31] scored varying degrees of bias ranging from “definitely high risk to definitely low risk of bias”. In these seven studies, the increase in scoring of “probably high risk of bias” was due to failure to report details of the study protocol (Table 1). Of these seven studies, four studies were at “definitely high risk of bias” for detection bias [27,31] or selective reporting bias [29,30]. As approximately two-thirds of the included studies were “definitely low risk of bias” to “probably high risk of bias”, the overall level of evidence level of this systematic review is moderate to low. All selected radiographic studies utilized baseline or control for comparison. However, there was limited quantitative data to enable a meaningful meta-analysis. The selected studies with controls were too heterogenous, and these studies utilized different lasers and had different treatment protocols and follow-up periods.

### 3.3. Study Characteristics

Of the 13 studies (Table 2), one was retrospective [30] and 12 were prospective [14,19,22,24,25,26,27,28,29,31,32,33]. Of the 12 prospective studies, eight were controlled trials [19,22,24,25,28,29,32,33]. Of the eight controlled trials, six were randomized [19,22,24,25,32,33]. The duration of the selected studies ranged from 3 months to 16 years. Four studies [26,28,29,31] reported implant loss during the duration of observation. Implant survival post-laser therapy reported in these four studies were 86.4% (19 of 22 implants) for up to a 3-year observation period [28], 96.0% (24 of 25 implants; for the one patient with two implants who dropped out after 3 months, the implant survival was unknown and was excluded from the calculation) for a 1-year observation period [31], 88.2% (15 of 17 implants) for a 12-year observation period [26], and 76.5% (13 of 17 implants in the laser and bone augmentation group) and 90.9% (20 of 22 implants in the laser and soft tissue resection group) for up to a 5-year observation period [29]. Two studies reported no implants were lost during the observation period and a 100% implant survival [22,32]. The remaining seven studies may have had 100% implant survival post-treatment as implant loss was not reported during the observation period. The sample size of the selected studies ranged from 10 patients to 68 patients. The age range of the patients was 20 to 85 years. The health status of the included patients was mostly not specified or systemically healthy. Other clinical parameters evaluated were: plaque index, bleeding on probing, probing pocket depth, suppuration, microbial analysis, width of keratinized tissue, peri-implant bone loss, and radiographic analysis.

Of the laser types evaluated in 13 studies (Table 3), two were diode (810 nm) [25,26], two were Nd:YAG (1064 nm) [24,30], six were Er:YAG (2940 nm) [19,22,27,31,32,33], and three were CO_2_ (10,600 nm) [14,28,29]. On the method of use, 8 of the 13 studies elevated a full-thickness flap before using the laser [14,26,27,28,29,31,32,33]. Cooling used during laser treatment was water for three studies [19,27,32], air and water for one study [24], or not specified for nine studies [14,22,25,26,28,29,30,31,33]. Nine studies [19,22,24,25,27,30,31,32,33] specified pulsed lasers, two specified continuous-wave laser emission [28,29], and two did not specify the emission mode [14,26]. Twelve studies [14,19,22,24,25,26,27,28,29,31,32,33] reported laser power or energy parameters, and one did not specify parameters [30]. Eight studies [14,19,24,25,26,28,29,33] specified laser irradiation exposure duration, and five did not specify duration [22,27,30,31,32]. Six studies [22,27,30,31,32,33] disclosed commercial support, four disclosed support from an educational institution or society [19,24,25,33], and four provided no disclosure [14,26,28,32].

Of the selected studies (Table 4), five had no control [14,26,27,30,31], two had controls that were non-surgical mechanical debridement [24,25], two had controls that were non-surgical mechanical and chemical debridement [19,32], two had controls that were decontamination with air-powder abrasives [22,28], one had a control that was soft tissue resection [29], and one had a control that was surgical regenerative therapy including mechanical debridement [33]. Before laser treatment, six studies [19,24,25,26,32,33] used nonsurgical mechanical intervention, one used systemic antimicrobial therapy [27], two used antimicrobial oral rinses [28,29], and four had no additional intervention [14,22,30,31]. Of the 13 studies, four had no conjunctive surgical therapy [19,22,24,25] and nine had surgical therapy in conjunction with laser therapy [14,26,27,29,31,32,33]. In addition to the laser treatment, seven studies [14,26,27,29,31,32,33] used bone grafting biomaterials, three did not mention biomaterials [19,22,28], and three did not use any grafting materials [24,25,30]. Of the selected studies, four reported use of systemic antibiotics [27,30,32,33], three reported pre-operative use of antimicrobial irrigant [26,28,29], four reported intra-operative use of antimicrobial irrigant [19,25,27,30], and six reported post-operative use of antimicrobial irrigant [19,27,30,31,32,33].

Implant types included in the studies included a wide range of manufacturers and different implant surfaces (Table 5). Four studies described the loading protocol after laser treatment [14,22,24,29], and this was not mentioned in the other nine studies [19,25,26,27,28,30,31,32,33]. Duration of implant function before peri-implantitis treatment ranged from 3 months to more than 15 years. The implant crowns were cemented in two studies [24,25], cemented or screw-retained in two studies [27,29], and method of retention was not mentioned in nine studies [14,19,22,26,28,30,31,32,33]. Occlusal adjustments were described in two studies [25,30], and were not mentioned [14,19,22,26,28,33] or not done in the other 11 studies [24,27,29,31,32]. Implant superstructures were removed in three studies [22,25,32], screw-retained prostheses were removed but cemented prostheses were left in situ in one study [29], and in the other nine studies, removal was either not mentioned or not done [14,19,24,26,27,28,30,31,33]. Implantoplasty was reported or shown in two studies [28,33], and was not mentioned [14,19,22,26] or not done [24,25,27,29,30,31,32] in the other 11 studies.

### 3.4. Primary Outcomes

With respect to radiographic assessment (Table 6 and Table 8), nine studies had radiographic standardization [22,24,25,27,28,29,31,32,33], and the remaining four did not mention or use standardization [14,19,26,30]. Five studies performed radiographic follow-up at 6 months [22,24,25,32,33], and the remaining eight studies did so at one year and later [14,19,26,27,28,29,30,31]. For radiographic outcome compared to baseline, three studies had statistically significant RBL gain [25,28,29], two reported no significant difference [22,24], and eight studies either did no statistical analysis or did not mention it [14,19,26,27,30,31,32,33]. As for radiographic outcome compared to control, two studies had significant RBL gain [28,29], four studies had no significant difference [19,22,24,33], one study had significant RBL loss [25], one study did not report statistical analysis [32], and five studies had no controls [14,26,27,30,31].

For RBL compared to baseline, the Nd:YAG laser had no significant effect in one study [24] and RBL gain in another study [30] with no statistical analysis; the diode laser had significant RBL loss in one study [25], and RBL gain in another study [26] where the significance was not analyzed; the Er:YAG laser did not significantly affect RBL in two studies [19,22] and in the other studies the RBL loss (one study) [32] or gain (three studies) [27,31,33] was not statistically analyzed; and the CO_2_ laser studies reported RBL gain that was not statistically analyzed [14,28,29]. Compared to control, the Nd:YAG (one study) [24] did not have a significant effect on the RBL; the diode laser had significant RBL loss in one study [25]; the Er:YAG laser did not significantly affect RBL in three studies [19,22,33], and in another study [32] the reduced RBL loss was not statistically analyzed; and the CO_2_ laser showed significant RBL gain in two studies [28,29] and no significant difference in another [29].

This systematic review, parsed by laser wavelength, revealed the following:

- For the two diode laser studies, one reported RBL gain compared to baseline [26], but the statistical significance was not analyzed. The other reported significant RBL loss compared to baseline and control [25].
- For the two Nd:YAG laser investigations, one showed RBL gain [30] compared to baseline, but without analysis of statistical significance. The other [24] indicated RBL loss compared to baseline and control that was not statistically significant.
- For the five Er:YAG laser studies, two reported RBL gain [27,31] compared to baseline but did not analyze the statistical significance of the change. One study [32] showed RBL loss compared to baseline and less RBL loss compared to control; the statistical significance of both results was not analyzed. Another reported RBL loss compared to baseline and control that was not significant [22]. One investigation reported no significant RBL change compared to either baseline or control [19]. Another study [33] reported RBL gain compared to control that was not significant, and RBL gain compared to baseline without analyzing the significance.
- For the three CO_2_ laser studies, two [28,29] reported RBL gain compared to baseline (statistical significance not analyzed) and significant RBL gain compared to control. The other study [14] reported RBL gain compared to baseline, but did not analyze the statistical significance.

Overall, the 13 studies revealed conflicting results for changes in bony defects. Eight studies showed evidence of RBL gain compared to baseline [14,26,27,28,29,30,31,33] and two showed evidence of RBL loss [25,32]. The statistical significance of the RBL changes was not analyzed in nine of these ten studies [14,26,27,28,29,30,31,32,33]. Three reported no statistically significant change from baseline [19,22,24].

Eight of 13 studies reported comparisons to control [19,22,24,25,28,29,32,33]. Of these eight studies, three showed RBL gain compared to control [28,29,33]; in two of these three studies RBL gain was statistically significant [28,29], and one was not significant [33]. The two studies [28,29] that showed statistically significant RBL gain compared to control were CO_2_ laser treatments compared to air abrasives by the same research group. As for the remaining five of these eight studies, two reported RBL loss that was not statistically significant [22,24], one reported no statistically significant RBL changes [19], one reported significant RBL loss [25], and one reported less RBL loss with no statistical analysis [32].

### 3.5. Secondary Outcomes

Comparing BOP to baseline (Table 7 and Table 8), six studies reported significant reduction [14,19,22,25,32,33], two analyzed significance but did not report it [24,27], and five did no statistical analysis [26,28,29,30,31]. Comparing BOP to control, six studies did statistical analysis [19,22,24,25,32,33], of which three reported significant BOP decrease [19,24,32], and three no difference [22,25,33]; of the remaining seven studies, five had no controls [14,26,27,30,31] and two provided no statistical analysis [28,29]. As for CAL compared to baseline, three studies reported significant improvement [19,28,33] and one reported attachment loss but no statistical analysis [29], and the remaining nine studies did not assess [24,25,27,31,32] or mention [14,22,26,30] it. As for CAL compared to control, of the four studies that did statistical analysis [19,28,29,33], two found significant improvement [28,29] and two did not find any difference [19,33]. Of the remaining nine studies, five had no control [14,26,27,30,31] and four did not evaluate or report [22,24,25,32]. As for PD compared to baseline, five studies reported statistically significant improvement [14,19,25,27,33]. Of the remaining eight studies [22,24,26,28,29,30,31,32], seven presented changes in PD but no statistical analysis was done or reported [22,24,28,29,30,31,32] and one did not assess PD [26]. As for PD compared to control, five studies reported statistical analysis [19,22,24,25,33], two showed significant improvement [24,33], and three reported no significant difference [19,22,25]. Of the remaining eight studies [14,26,27,28,29,30,31,32], five had no controls [14,26,27,30,31], two did no statistical analysis [28,29], and one did not mention [32]. Two studies conducted a microbial analysis: one study reported almost complete elimination of Porphyromonas gingivalis (Pg) [26] and one did not find a significant difference [25]. For the remaining 11 studies, microbial analysis was not done or mentioned. As for adverse reactions, two studies reported no adverse reactions [25,28], four reported some minor adverse reactions [14,19,22,29], one study reported that membrane exposure significantly reduced PD reduction and CAL gain [33], and the remaining six studies did not mention [24,26,27,30,31,32].

The clinical significance of laser therapy using different lasers is described in Table 8. Laser therapy was compared to baseline or control. Control was either mechanical debridement with curettes or air-powder abrasives.

Inflammation was evaluated via BOP, sulcus bleeding index (SBI), or suppuration. Compared to baseline, the Nd:YAG laser reduced inflammation in one study [24], although the significance was not analyzed; the diode laser had no significant effect on inflammation as reported in one study [25]; the Er:YAG laser significantly reduced inflammation in four studies [19,22,32,33] and in one study the reduction was not statistically analyzed [31]; and for the CO_2_ laser, inflammation was significantly reduced in one study [14], and in two studies [28,29] the increase in inflammation was not statistically analyzed. The remaining three studies did not report inflammatory parameters [26,27,30]. Compared to control, the Nd:YAG (one study) [24] and the diode laser (one study) [25] did not have a significant effect on inflammation; the Er:YAG significantly reduced inflammation in two studies [19,32], and was not statistically significant in two studies [22,33]; and for the CO_2_ lasers, the increase in inflammation in one study [28] was not statistically analyzed, and in one other study [29] the increase in the residual bone group or the decrease in the augmented bone group was not statistically analyzed.

For PD compared to baseline, the Nd:YAG laser reduced PD in one study [24] with no reported statistical analysis; the diode laser significantly reduced the PD in one study [25]; the Er:YAG laser significantly reduced PD in three studies [19,27,33] and in three studies [22,31,32] the reduction was not statistically analyzed; and for the CO_2_ laser, PD was significantly reduced in one study [14], and in two studies [28,29] the increase was not statistically analyzed. Compared to control, the Nd:YAG (one study) [24] and the diode laser (one study) [25] did not have a significant effect on the PD; the Er:YAG laser significantly reduced PD in one study [33], did not significantly affect PD in two studies [19,22], and in another study [32] the reduction was not statistically analyzed; and for the CO_2_ laser, the reduction in PD in two studies [28,29] and the insignificant change in PD in one study [29] were not statistically analyzed.

## 4. Discussion

Periodontal regeneration, defined by the American Academy of Periodontology (AAP) and published by several investigators [58], is the restoration of lost or diminished periodontal tissues including cementum, periodontal ligament, and alveolar bone. Human histological studies are the only way to assess periodontal regeneration. Osseointegrated dental implants lack cementum and periodontal ligament, so a direct comparison between teeth and implants is not possible. Histological evaluation of regeneration has been the most accurate way to evaluate regeneration around teeth [59,60]. To date, few clinical studies have reported histological outcomes after laser treatment of peri-implantitis, and these were conducted in dogs [61,62]; therefore, RBL changes post-laser treatment may be the next available option to infer histologic changes. Radiographic evaluation of bone fill and increase in radio-opacity post-treatment may indicate regeneration or repair and may be a possible way to infer regeneration or repair when bone grafting material is not used in conjunction with the laser treatment. The selected studies in this systematic review are focused on the radiographic methodology and post-treatment changes to evaluate whether laser treatment can provide positive outcomes. A recent systematic review and meta-analysis on laser treatment of peri-implantitis reported only three studies [22,25,29] for RBL changes using high-intensity laser therapy [63]. These three studies are included in the 13 studies analyzed in this review.

Positive radiographic interpretation can be bone fill around implants after peri-implantitis treatment. Radiographic determination of bone changes around implants and teeth can be limited by non-standardized radiologic methodology with inconsistent sensor angulations, position, and sensitivity [64]. In some of the included studies, efforts to standardize radiographs were not mentioned [14,19,30] or done. In addition, methods to assess bone gain or loss were different in different studies.

Clinician interpretation of radiographs can be subjective and biased. The level of expertise of the clinician when taking or interpreting radiographs may vary from radiologist, dentist, or dental student, thus affecting the accuracy and consistency of the interpretation. Computer software-assisted radiographic assessment can be reproducible and reduce operator bias and inter-operator discrepancy [64,65]. However, not all the selected studies used software. The use of software is also not without limitations. The accuracy of software is dependent on operator calibration of the computer to a fixed structure in the mouth; thus, operator errors or calibration errors while using the software can also limit the accuracy of the results.

Radiographic evaluation can be limited by inter-patient variations. Different patients may have different rates and degrees of osseous healing and radio-opacity. In addition, different patients may have different bone and tissue density that may absorb radiation differently [66]: even within the same patient, slight changes in tissue remodeling at pre-treatment and post-treatment time points may affect the exact comparison of radiographs [67]. The time points at which the radiographs were taken may also have an impact on the radio-opacity of the bone fill. When radiographic evaluation is done too early (1 to 3 months), it may provide an erroneous impression that bone fill was not significant. Moreover, documented studies on the degree of calcification of bone before it becomes radiographically apparent have reported time intervals of at least 6 months post-therapy [68]. Most studies were not clear as to which time interval would best reflect bone fill, and in some cases, non-significant results may be the result of insufficient time allocated for the bone changes to be mineralized adequately to show radiographically. In addition, most of the selected studies have inconsistent follow-up time intervals and missing radiographic evaluation at certain follow-up intervals.

The clinical effects of laser treatment at more than 6 months also show promise for radiographic outcomes, probing pocket depth changes, and control of inflammation, as most of the selected studies reported reduction in PD [14,19,22,24,25,27,28,29,31,32,33] and inflammation [14,19,22,24,29,31,33] compared to baseline (Table 8). When compared to control, many of the selected studies with controls reported positive radiographic outcomes [28,29,33], probing depth [28,29,32,33], and inflammation reduction [19,32,33], and that laser peri-implantitis treatment was as good as or possibly better than control. However, because significance was not analyzed in most of these studies, the results can only suggest a positive outcome but cannot definitively conclude that outcome is indeed statistically significant.

The risk of bias of the included studies was variable. A quarter of the studies showed definitely or probably low risk of bias; the rest were mixed, with approximately a third of the studies showing 1–2 points at definitely high risk of bias. However, the assessment of the risk of bias alone may not be sufficient to fully assess the body of evidence. The quality of evidence can be compromised by a number of potential biases. For example, 8 of the 13 studies either did not include or report on the statistical significance of radiographic bone level changes, thus showing a level of possible reporting bias [14,19,26,27,30,31,32,33]. Only two-thirds of the six randomized controlled trials included in this systematic review calculated the number of patients required for an adequately powered trial [22,24,25,33], thus revealing a potential imprecision bias in the other two trials [19,32]. A commercial bias may apply to 10 of the 13 studies that either reported some degree of industry sponsorship [22,27,29,30,31,33] or provided no disclosure [14,26,28,32].

A possible limitation of the review process was that the keywords used in the search may have excluded articles published in a foreign language, hence some pertinent articles may have been missed.

The evidence presented in this systematic review was also constrained by insufficient standardization of data reported in the selected articles. This shortcoming can lead to confounding factors that may influence the results of this review. In addition, variability in the detected bias among the chosen papers further limited the strength of the data synthesis. Nevertheless, this review accurately reported the variables identified in the studies in order to establish a baseline of understanding of how adjunctive laser use during treatment of peri-implantitis may affect radiographic bone level changes.

Conventional surgical therapies are demanding, technique-sensitive, and time consuming. Laser therapies may reduce clinician fatigue and stress while resulting in positive clinical outcomes. Further research studies will provide more tangible clinical data on the specific type of lasers and their associated clinical outcomes.

### 4.1. Recommendations for Laser Treatment Protocols

For the treatment for peri-implantitis with dental lasers, the researcher and clinician should consider laser treatment protocols that have shown evidence of the following: (1) laser reduction of infection, peri-implant bacteria, or viruses; (2) laser reduction of inflammation or inflammatory cytokines; (3) minimal tissue necrosis; (4) biostimulatory or enhanced laser-induced healing; and (5) consideration for adjunctive non-laser (mechanical debridement, air abrasives, or topical chemical agents) and laser approaches for implant rescue. To ensure safe use of the laser for patient treatment, the clinician should be well educated in dental lasers and abide by the laser guidelines and protocols of the manufacturer.

### 4.2. Recommendations for Future Studies

Recommendations for future research should include careful documentation of all collected data (Table 9) to facilitate meta-analyses of systematic reviews. In the conduct of a study, every attempt should be made to evaluate for statistical significance.

Table 9 was specifically devised as a suggested guideline to enable future investigators to: (1) consider the range of variables applicable to laser-based peri-implantitis treatment, (2) develop more consistent study designs with greater reproducibility, (3) improve standardization in data collection, (4) increase the validity of research findings, (5) reduce occurrences of bias, and (6) assure greater relevance and translation of research findings to the clinician.

## 5. Conclusions

The statistical significance of the RBL changes was not analyzed in most of the 13 studies; therefore, definitive RBL gain remains inconclusive. However, the use of dental lasers to encourage radiographic bone fill may show some promise, as most studies reported bone gain compared to baseline or control. The following conclusions about dental lasers in the treatment of peri-implantitis are within the limits of this systematic review: (1) laser treatment may enhance bone gain in peri-implantitis defects, (2) laser treatment may reduce BOP and PDs, and (3) laser peri-implantitis treatment may be as good as if not better than mechanical debridement or air abrasives. Unfortunately, definitive conclusions can only be made with proper statistical analysis of the bone level changes, which was lacking in the currently available studies. Further studies with an emphasis on supporting statistics are needed. Table 9 outlines the research data needed to aid future systematic reviews on laser treatment of peri-implantitis.

## Figures and Tables

**Table 1:** OHAT risk of bias analysis.

OHAT Domain and Questions	Abduljabbar et al., 2017 [24]	Arısan et al., 2015 [25]	Bach 2009 [26]	Clem and Gunsolley 2019 [27]	Deppe et al., 2005 [28]	Deppe et al., 2007 [29]	Nicholson et al., 2014 [30]	Norton 2017 [31]	Peng and Tomov 2012 [32]	Renvert et al., 2011 [22]	Romanos et al., 2008 [14]	Schwarz et al., 2006 [19]	Wang et al., 2020 [33]
**Selection Bias**													
1. Was administered dose or exposure duration level adequately randomized?	++	++	N/A	N/A	N/A	N/A	N/A	N/A	++	++	N/A	++	++
2. Was allocation to study groups adequately concealed?	+	NR	N/A	N/A	N/A	N/A	N/A	N/A	NR	++	N/A	NR	++
3. Did the selection of study participants result in appropriate comparison groups?	N/A	N/A	+	+	NR	NR	+	NR	N/A	N/A	+	N/A	N/A
**Confounding Bias**													
4. Did the study design or analysis account for important confounding and modifying variables?	N/A	N/A	NR	NR	NR	NR	NR	+	N/A	N/A	NR	N/A	N/A
**Performance Bias**													
5. Were the research personnel and human subjects blinded to the study group during the study?	+	NR	N/A	N/A	N/A	N/A	N/A	N/A	NR	++	N/A	NR	++
**Attrition/Exclusion Bias**													
6. Were outcome data complete without attrition or exclusion from the analysis?	++	++	++	++	−	−	+	+	++	++	++	++	++
**Detection Bias**													
7. Can we be confident in the exposure characterization?	++	++	++	++	++	++	NR	NR	++	++	NR	++	++
8. Can we be confident in the outcome assessment?	++	NR	NR	−−	NR	NR	NR	−−	NR	++	NR	NR	++
**Selective Reporting Bias**													
9. Were all measured outcomes reported?	+	++	++	++	++	−−	−−	++	+	++	++	++	++
**Other Bias**													
10. Were statistical methods appropriate?	++	++	NR	−−	++	++	NR	NR	++	++	NR	++	++
11. Did researchers adhere to the study protocol?	+	+	+	+	+	+	+	+	+	+	+	+	+
12. Did the study design or analysis account for important confounding and modifying variables (including unintended co-exposures) in experimental studies?	+	+	N/A	N/A	N/A	N/A	N/A	N/A	+	+	N/A	+	+

++ Definitely low risk of bias, + probably low risk of bias, NR not reported, − probably high risk of bias, −− definitely high risk of bias, N/A means a particular OHAT question does not apply.

**Table 2:** Study design and details.

Study	Study Design	Duration	Follow-Up	Sample Size	Gender	Age Range (Mean)	Health Status	Clinical Parameters
Abduljabbar et al., 2017 [24]	Prospective, parallel design, single-blinded, randomized, controlled trial	6 mos	3 mos 6 mos	63 patients: 32 control gp: nonsurgical mechanical debridement [MD], 39 implants 31 laser gp: MD and single application of Nd:YAG laser, 35 implants	63 males	Group 1 (MD): 31–58 yrs (43.6 yrs) Group 2 (MD + laser): 29–60 yrs (40.5 yrs)	Systemically healthy, no smokers	Plaque Index (PI) Bleeding on probing (BOP) Probing depth (PD) Suppuration Peri-implant crestal bone loss Radiographic analysis
Arısan et al., 2015 [25]	Prospective, parallel design, split-mouth, randomized, controlled trial	6 mos, February 2010 to May 2013	1 mo 6 mos	10 patients: 5 control gp: MD, 24 implants 5 laser gp: MD and single application of 810-nm diode laser, 24 implants	3 males 7 females	54–76 yrs (55.1 yrs)	Systemically healthy, no smokers	PI BOP PD Marginal bone loss Microbial analysis Radiographic analysis
Bach 2009 [26]	Prospective, longitudinal study	12 yrs, 1995–2007	4 wks 6 mos 1 yr Every yr	10 patients, 17 implants	5 males 5 females	20–70 yrs	Not specified	Microbial analysis Radiographic analysis
Clem and Gunsolley 2019 [27]	Prospective, consecutive, longitudinal study	2+ yrs, June 2014 to November 2016	6 mos 12 mos	20 patients, 23 implants	11 males 9 females	56–85 yrs	Systemically healthy except for: Type II controlled diabetes: 2 Cardiovascular disease: 8 Bisphosphonates therapy: 2 Self-reported smoker: 1	PD Implant bone levels and fill Radiographic analysis
Deppe et al., 2005 [28]	Prospective, controlled, longitudinal study	3 yrs, February 1999 to February 2002	4 mos 6–38 mos (mean 17 mos)	16 patients: 6 control gp: air-powder abrasive [APA], 19 implants 10 laser gp: APA and single application of CO_2_ laser, 22 implants	Not specified	Not specified	Not specified	PI BOP PD Distance between implant shoulder and marginal mucosa (DIM) Attachment level (PD + DIM) Radiographic analysis of distance between implant and bone (DIB)
Deppe et al., 2007 [29]	Prospective, controlled, longitudinal study	5+ yrs, January 1999–May 2004	5–59 mos (mean 37 mos)	32 patients: 13 control gp: air-powder abrasive [APA], 34 implants 19 laser gp: APA and single application of CO_2_ laser, 39 implants	Not specified	Not specified	Not specified	PI BOP PD Distance between implant shoulder and mucosa (DIM) Clinical attachment level (CAL) Distance from implant shoulder to first bone contact (DIB) Radiographic analysis
Nicholson et al., 2014 [30]	Retrospective longitudinal study	3 mos–16 yrs	2 mos 8 mos 36 mos 48 mos	16 patients, number of implants not specified	7 males 9 females	32–79 yrs (54 yrs)	Not specified	Radiographic analysis
Norton 2017 [31]	Prospective, longitudinal study	1+ yrs, October 2013–February 2015	1 yr	20 patients, 27 implants	Not specified	Not specified	Smoking did not preclude inclusion	BOP PD Suppuration Radiographic analysis
Peng and Tomov 2012 [32]	Prospective, parallel design, single-blinded, randomized, controlled trial	1 yr, September 2010 to August 2011	6 mos	68 patients, 128 implants Mechanical therapy gp (number of patients not specified) Laser therapy gp (number of patients not specified)	Not specified	Not specified	Not specified	BOP PD Radiographic analysis
Renvert et al., 2011 [22]	Prospective, parallel design, single-blinded, randomized, controlled trial	2 yrs, October 2007 to September 2009	6 mos	42 patients: 21 air abrasive gp: 45 implants 21 laser gp: 55 implants	Not specified	(Control: 68.9 yrs, Laser: 68.5 yrs)	Not specified	BOP PD Suppuration Radiographic analysis
Romanos et al., 2008 [14]	Prospective longitudinal study	27.10 mos (±17.83)	1 mo 3 mos 6 mos 9 mos then every year	15 patients, 19 implants	5 males 10 females	(57.21 yrs)	Not specified	PI BOP PD Width of keratinized tissue Bone loss Radiographic analysis of bone fill
Schwarz et al., 2006 [19]	Prospective, parallel design, randomized, controlled trial	12 mos	3 mos 6 mos 12 mos	20 patients: 10 control gp: mechanical debridement, 20 implants 10 laser gp: 20 implants	Control: 5 males 5 females Laser: 4 males 6 females	(Control: 52 yrs, Laser: 56 yrs)	No systemic diseases Patients who smoked occasionally were not categorized as smokers	PI BOP PD Gingival recession CAL Radiographic analysis
Wang et al., 2020 [33]	Prospective, parallel design, double-blinded, randomized, controlled trial	24 wks, June 2017 to November 2018	24 wks	24 patients: 12 control gp: open flap mechanical debridement, bone grafting and membrane, 12 implants 12 laser gp: 12 implants	Control: 7 males 5 females Laser: 7 males 5 females	(Control: 63.41 yrs, Laser: 66.41 yrs)	American Society of Anesthesiologists (ASA) I or II Not on medications modifying bone metabolism No smokers or smokers who quit <6 mos	PI BOP PD GR CAL Gingival index Radiographic analysis of bone fillRadiographic analysis of linear bone gain

PI: plaque index, GR: gingival recession, BOP: bleeding on probing, PD: probing depth, CAL: clinical attachment level.

**Table 3:** Laser details and protocol.

Study	Type of Laser	Manufacturer and Model	Beam Delivery System	Cooling during Laser Treatment	Laser Parameters	Method of Laser Use	Disclosure and Commercial Support
Abduljabbar et al., 2017 [24]	Nd:YAG, 1064 nm	Genius Dental, Tureby, Denmark	300-micron optical fiber	Air and water cooling	4.0 W, 80 mJ per pulse, 50 Hz pulse rate, 350-ms pulse width	After mechanical debridement with plastic curette, 300-micron fiber inserted into peri-implant pocket almost parallel to the implant, then moved in a mesial-distal direction for 60 to 120 s	Research group funded by Deanship of Scientific Research at King Saud University, Riyadh, Saudi Arabia
Arısan et al., 2015 [25]	Diode, 810 nm	Denlase 810/7, Beijing, China	Standard, uninitiated 400-micron optical fiber tip	Not specified	1.0 W, pulsed mode, 3 J/cm^2^, 400 mW/cm^2^, 1.5 J	After mechanical debridement with plastic curette, uninitiated tip inserted parallel to the long axis of the implant, about 1 mm from the most apical level of the peri-implant sulci Tip moved in a mesiodistal and apicocoronal direction around the implant for 60 s Laser spot diameter 1 mm	Study supported by a grant from the Istanbul University Research Fund
Bach 2009 [26]	Diode, 810 nm	Oralia 01 IST, Constance, Germany	Optical fiber, contact	Not specified	1.0 W, emission mode not specified	After mucoperiosteal flap and removal of granulation tissue, decontamination for 20 s	Not specified
Clem and Gunsolley 2019 [27]	Er:YAG, 2940 nm	J. Morita AdvErL EVO, Osaka, Japan	Radial firing tipWorking distance not specified	Sterile water 5 mL/min	50 mJ/mm^2^, 20 Hz	After full-thickness mucoperiosteal flap, granulomatous tissue within defects removed with laser, then implant surfaces irradiated with at least two complete passes or until a change in the reflective quality of the implant surface or dark gray discoloration of the implant surface was observed	J Morita Corp. provided laser equipment and support for creation of manuscript Study partially supported by an educational grant from J Morita Corp.
Deppe et al., 2005 [28]	CO_2_, 10,600 nm	DEKA 20C, Freising, Germany	Articulated arm and handpiece with focus distance of 125 mm, working distance not specified	Not specified	CW, 2.5 W	After full-thickness flap, granulation tissue removal, and air-powder treatment of implant surfaces for 60 s, implant decontamination for 12 × 5 s laser irradiation. Laser spot diameter 200 microns when focused	Not specified
Deppe et al., 2007 [29]	CO_2_, 10,600 nm	DEKA 20C with Swiftlase scanner, Freising, Germany	Articulated arm, scanner and handpiece focal length 125 mm, spot diameter Working distance not specified Angled mirrors (90 and 120 degrees) mounted on handpiece	Not specified	CW, 2.5 W	After full-thickness flap, implant decontamination for 12 × 5 s laser irradiation Laser spot diameter 200 microns when focused Scanner used in CW mode, energy density of 175 J/cm^2^, exposure time 5 s, to reduce local heat accumulation by sweeping a focused CO_2_ laser beam in 0.1 s over an area of 3.0 mm diameter, (resulting in a total of 7.06 mm^2^). Dwell time on each point was less than 1 ms	Research project supported by Friadent
Nicholson et al., 2014 [30]	Nd:YAG, 1064 nm	Millennium Dental Technologies PerioLase MVP-7, Cerritos, California	Optical fiber	Not specified	Not specified Light dosage about one-third the energy applied around teeth	Laser first used to remove inflamed pocket epithelium, open the pocket for access, and decontaminate implant After removal of accretions from implant surface with hand instruments and ultrasonic scaler, and after decortication, laser then used to form a stable fibrin clot	Study supported by Millennium Dental Technologies One author received consulting fees from Millennium, 3 authors are principals of Millennium Manuscript represented the best submitted cases from responders
Norton 2017 [31]	Er:YAG, 2940 nm	J. Morita AdvErL EVO, Osaka, Japan	Tip not specified Working distance not specified	Not specified	Initial settings of 50 mJ, 25 Hz	After flap reflection and removal of fibrous tissue capsule surrounding the implants and gross hard deposits with curettes, contaminated implant surfaces treated with laser. Settings were occasionally varied according to need to ensure comprehensively debrided, cleaned, and decontaminated implant surfaces	Study funded by a grant from Morita, Inc.
Peng and Tomov 2012 [32]	Er:YAG, 2940 nm	Syneron LiteTouch, Yokneam Illit, Israel	Laser-in-handpiece, 1300-micron tip, contact or noncontact (working distance 1.5 mm) Flap incision: Contact, 0.4 × 17 mm tip Granulation tissue ablation: Noncontact, 1.3 × 14 mm tip Bone remodeling: Noncontact, 1.3 × 19 mm tip Implant decontamination: Noncontact, 1.3 × 17 mm tip Decortication: Noncontact, 1.3 × 19 mm tip	Water spray levels settings 5–8, depending on procedure: Flap incision: 5–6 Granulation tissue ablation: 6 Bone remodeling: 8 Implant decontamination: 6 Decortication: 8	Flap incision: 200 mJ, 35 Hz Granulation tissue ablation: 400 mJ, 17 Hz Bone remodeling: 300 mJ, 25 Hz Implant decontamination: 150 mJ, 45 Hz Decortication: 300 mJ, 25 Hz	Flap incision with laser, reflection, noncontact tip to remove granulation tissue, and clean implant surface by systematically moving tip along surface Laser tip in constant motion	Not specified
Renvert et al., 2011 [22]	Er:YAG. 2940 nm	KaVo Key Laser 3, Biberach, Germany	Cone-shaped sapphire tip, working distance not specified	Not specified	100 mJ/pulse, 10 Hz (12.7 J/cm^2^)	Tip used in parallel mode using a semicircular motion around the circumferential pocket	Study sponsored by EMS, KAVO, Philips Oral Healthcare
Romanos et al., 2008 [14]	CO_2_, 10,600 nm	Weil Dental SC 20 or DEKA Smart US-20D, Freising, Germany	Articulated arm and handpiece, noncontact Working distance not specified Tip not specified	Not specified	2.84 ± 0.83 W. Emission mode not specified	After full-thickness mucoperiosteal flap, granulomatous tissue removed with titanium curettes, and exposed implant surfaces irradiated for 1 min	Not specified
Schwarz et al., 2006 [19]	Er:YAG, 2940 nm	KaVo KEY 3, Biberach, Germany	Specially designed periodontal handpiece, and cone-shaped glass fiber tip emitting a radial and axial laser beam, contact	Water irrigation	10 Hz, 100 mJ/pulse (12.7 J/cm^2^), pulse energy at tip approximately 85 mJ/pulse	Semicircular motion from coronal to apical parallel to implant surface Both control and laser: 6 min avg per implant	Study supported by grant from Arbeitsgemeinschaft für Kieferchirurgie innerhalb der Deutschen Gesselschaft für Zahn-, Mund- und Kieferheilkunde
Wang et al., 2020 [33]	Er:YAG, 2940 nm	Morita AdvErL EVO, Kyoto, Japan	Optical transmission cable with metal-shelf tips PS600T, PSM600T, R600T	Not specified	50 mJ/pulse, 25 Hz, 0.5 mm/s for granulation tissue removal and implant decontamination 30 mJ/pulse, 20 Hz, 0.5 mm/s for irradiation of implant defect and tissue	Debridement and surface decontamination of implant surfaces and removal of inflamed tissue with laser Slow linear motion of 0.5 mm/s vertically and horizontally for 3–5 min After implantoplasty for peri-implant suprabony defect, infrabony defect debrided with laser	Study supported by grants from J Morita (Tokyo, Japan) and University of Michigan School of Dentistry Department of Periodontics and Oral Medical Clinical Research Fund BioHorizons (Birmingham, Alabama) provided biomaterials Co-author Wang HL has lectured for J Morita and received honoraria

**Table 4:** Clinical therapy.

Study	Control	Non-Surgical Intervention before Laser Treatment	Surgical Therapy in Conjunction with Laser Treatment	Use of Biomaterials	Use of Antibiotics	Use of Oral Irrigant	Follow-Up Care
Abdul-jabbar et al., 2017 [24]	Nonsurgical mechanical debridement using plastic curettes	Nonsurgical mechanical debridement using plastic curettes, plaque removed from implant surfaces	No surgical therapy	Not used	Not used	Not used	Not mentioned
Arısan et al., 2015 [25]	Nonsurgical mechanical debridement using plastic curettes	Nonsurgical mechanical debridement using plastic curettes Supragingival plaque removed by sterile gauze	No surgical therapy	Not used	Not used	Intraoperative: Peri-implant sulci of all implants were washed with sterile saline solution to remove debris	Not mentioned
Bach 2009 [26]	No control	Cleaning and polishing	Mucoperiosteal flap, removal of granulation tissue, decontamination with diode laser, soft tissues apically positioned. Bone augmentation and mucogingival corrections when needed	Materials used for bone augmentation not specified	Not mentioned	Preoperative: Application of disinfecting agents	4 wks, 6 mos, 1 yr, and annually: exposed implant surfaces decontaminated with diode laser
Clem and Gunsolley 2019 [27]	No control	Antimicrobial therapy starting the day before surgery	Full thickness mucoperiosteal flaps, laser removal of granulomatous tissue. Implant surfaces irradiated. Bone grafting for vertical defects	Patient received one of the three options: (1) mineralized freeze-dried bone allograft (FDBA; creos™ allo.gain, Nobel Biocare) with recombinant human platelet-derived growth factor-BB (rhPDGF-BB; creos™ allo.gain, Nobel Biocare) (2) 70/30 mix of mineralized FDBA/demineralized FDBA (DFDBA) with PDGF (OraGraft^®^ DGC1, LifeNet Health) with rhPDGF-BB (GEM 21S^®^, Lynch Biologics) (3) DFDBA and enamel matrix derivative (EMD; Emdogain^®^, Straumann) Rapidly absorbing collagen membrane used only when facial or lingual defects were present	Metronidazole 500 mg and amoxicillin 500 mg for 10 days bid starting the day before surgery	Intraoperative: H_2_O_2_ soaked gauze in the defects for about 10 s and irrigated with sterile saline Postoperative: Chlorhexidine 0.12% swabs	2 wks: Patients to use soft toothbrush and light dental tape 4 wks: Reinforced oral hygiene instruction on more aggressive brushing or a modified plaque control approach
Deppe et al., 2005 [28]	Conventional decontamination with air-powder abrasive	Chlorhexidine 0.3% for 3 weeks before treatment	Both groups: full thickness flaps, granulation tissue removal, implant decontamination, implant surfaces treated with air-powder-abrasive, then flaps resected, re-positioned, and sutured	Not mentioned	Not used	Preoperative: Chlorhexidine 0.3%	Not mentioned
Deppe et al., 2007 [29]	Soft tissue resection after conventional decontamination	Chlorhexidine 0.3% for 3 weeks before treatment	Full-thickness flaps, granulation tissue removal and implant decontamination, bone augmentation when recommended, then flaps resected, re-positioned, and sutured	Bone augmentation recommended only for screw-retained prosthetics 50–50 mix of resorbable beta-tricalcium phosphate (βTCP or Cerasorb^®^, Curasan) with bone harvested from mandibular retromolar region Implants were submerged and covered by nonresorbable membrane (GORE-TEX^®^ G 4, W. L. Gore)	Not used	Preoperative: Chlorhexidine 0.3%	Not mentioned
Nicholson et al., 2014 [30]	No control	None	Surgical therapy in conjunction with laser treatment as part of the LAPIP protocol	No biomaterials used	Antimicrobial therapy post-treatment as part of the LAPIP protocol	Intraoperative: 0.12% chlorohexidine as part of the LAPIP protocol Postoperative: 0.12% chlorohexidine as part of the LAPIP protocol	Not mentioned
Norton 2017 [31]	No control	None	Open flap surgical debridement, fibrous tissue and hard deposits removed using curettes, laser implant decontamination, regeneration therapy with bone graft and membrane, flap sutured	Regenerative Therapy: Defects grafted with anorganic bovine bone mineral, rehydrated in sterile saline (Bio-Oss^®^, Geistlich) and confined by use of a resorbable collagen barrier membrane (Bio-Gide^®^, Geistlich) fixed in position with titanium tacks (FRIOS, Dentsply)	No antibiotics were prescribed	Intraoperative: No chlorohexidine used Postoperative: Chlorhexidine 0.2% mouthrinse, 10 mL twice a day for 1 min for 1 wk	Not mentioned
Peng and Tomov 2012 [32]	Conventional mechanical therapy with sharp curettes and ultrasonic device, followed by chemical debridement with tetracycline solution	Nonsurgical hygiene phase to reduce inflammation	Flap raised to access implant surface, granulation tissue removed with laser, laser in noncontact mode if calculus, rinsed with sterile saline, bone augmentation when required	Bone augmentation when required with deproteinized bovine bone (Bio-Oss^®^, Gesitlich) and bone allograft (Dembone^®^) with or without an absorbable biomembrane, material not specified	Clindamycin 150 mg, 50 tabs and Antibacterial periodontal treatment was repeated if inflammation recurred	Postoperative: Chlorhexidine 0.2% starting the next day for two weeks three times per day	Supportive phase to maintain long-term results. Inflammation detected on recall visit was treated with repeated antibacterial periodontal treatment
Renvert et al., 2011 [22]	Non-surgical debridement with air-abrasive device	None	No surgical therapy	Not mentioned	Not mentioned	Not mentioned	At all study time points, patients received individualized oral hygiene instructions. After 3 mos: Patients also received a sonic toothbrush and additional brush heads
Romanos et al., 2008 [14]	No control	None	Full-thickness mucoperiosteal flap elevated, granulomatous tissue removed with titanium curettes, laser was used, flaps were sutured	10 bony lesions were augmented with autogenous bone 9 defects with cancellous bone graft (Bio-Oss^®^, Osteohealth) Augmented sites were covered with collagen membranes (Bio-Gide^®^, Osteohealth) fixed with titanium pins (FRIOS^®^, FRIADENT) Mucoperiosteal flaps were closed with 4-0 silk sutures (RESORBA^®^)	Not used	Not mentioned	Not mentioned
Schwarz et al., 2006 [19]	Nonsurgical mechanical debridement using plastic curettes and antiseptic therapy	All patients: For 2 weeks before treatment, supragingival professional implant/tooth cleaning using rubber cups and polishing paste and oral hygiene instructions Patients with chronic periodontitis: Additional scaling and root planing using hand instruments	No surgical therapy	Not mentioned	Not mentioned	Intraoperative: In control only, pocket irrigation with 0.2% chlorhexidine digluconate solution, then subgingival application of 0.2% chlorohexidine gel Postoperative: Chlorohexidine rinse twice a day for 2 min for 2 weeks	In control group: Chlorohexidine rinse twice a day for 2 wks post-treatment Both groups: Supragingival professional implant/tooth cleaning and oral hygiene also at baseline, 1, 3, 6, 12 mos Both groups: Due to increased BOP and CAL, all patients were discontinued from study at 12 mos, and treated with Er:YAG laser therapy and bone augmentation
Wang et al., 2020 [33]	Surgical regenerative therapy including mechanical debridement and guided bone regeneration same as test gp, but no laser therapy	Full mouth prophylaxis or periodontal maintenance with piezo-instruments and stainless-steel hand scalers without subgingival implant debridement	Both test and control groups received open flap mechanical debridement, supracrestal implantoplasty, bone grafting, and acellular dermal matrix membrane Laser group used Er:YAG laser to modulate and remove inflammatory tissue and decontaminate implant surfaces	Bone grafting and regenerative therapy of infrabony defects. Mineralized bone allograft used in both groups Composite graft included 3:1 ratio of allograft and demineralized bone fibers (MinerOss and Grafton, BioHorizons) Absorbable acellular dermal matrix (ADM) membrane (Alloderm GBR, BioHorizons) was used Flap was sutured with polytetrafluorethylene (PTFE) sutures (Cytoplast, BioHorizons) Sutures were left for at least 14 days A periodontal dressing (Coe-Pak Periodontal Dressing, Patterson Dental) was used	Postoperatively, all patients were prescribed 500 mg Amoxicillin tid for 10 days; if patients were allergic, Azithromycin 500 mg for the first day and 250 mg for the next 3 days Ibuprofen 600 nm as needed for pain control	Postoperative: Chlorohexidine rinse twice a day for 1 min, bid for 1 week	Patients in both groups avoided brushing or touching the operated area for 2 weeks. 3 and 6 mos: Maintenance was performed All patients completed the 6 mos clinical trial and follow-up

**Table 5:** Implant details and restorative management.

Study	Number of Implant and Implant Type	Loading Protocol Initial Placement or after Peri-Implant Treatment	Duration of Implant Function before Treatment [Mean (Range)]	Implant-Restoration Connection	Occlusal Adjustments	Implant Crown Removed during Treatment	Implantoplasty in Conjunction with Laser Treatment
Abdul-jabbar et al., 2017 [24]	Group 1 (MD): 39 platform-switched Straumann^®^ Bone Level implants Group 2 (laser): 35 platform-switched Straumann^®^ Bone Level implants	Delayed-loaded: Loaded about 4 mos after initial implant placement	Group 1: 4.4 yrs (2–6.5 yrs) Group 2: 4.8 years (1–5.3 yrs)	All implants: Cement-retained	Not done	Not done	Not done
Arısan et al., 2015 [25]	Two-piece, tapered root form, rough surface (acid etched and sand blasted) implants: 48 (15 MIS^®^, 12 CAMLOG Biotechnologies, 8 Nobel Biocare™ Replace^®^, 7 BioHorizons^®^, 6 not mentioned)	Not mentioned	19.4 mos (12.2–25.2 mos)	All implants:Cement-retained	Occlusal contacts were checked to ensure the absence of overloading	All superstructures were removed, then recemented after treatment with a polycarboxylate cement An acrylic-based temporary crown was cemented on the treated implants if the permanent restoration was faulty	Not done
Bach 2009 [26]	17 implants: - implant details not specified - 2 implants lost in the 12 yr period	Not mentioned	Not mentioned	Not mentioned	Not mentioned	Not mentioned	Not mentioned
Clem and Gunsolley 2019 [27]	Enhanced titanium surface implants: 17 Machined surface implants: 6	Not mentioned	14 of 23 implants in function >5 yrs Implants in function: 6 < 5 yrs 9 > 5 yrs 3 > 10 yrs 2 > 15 yrs	11 stock-cemented 7 custom-cemented 2 screw-retained	Not done	Not done	Not done
Deppe et al., 2005 [28]	Group 1 (control): 19 (17 IMZ^®^, 2 Frialit 2^®^) - 3 implants lost Group 2 (laser): 22 (13 IMZ^®^, 4 Frialit 2^®^, 2 Brånemark^®^, 3 ITI-screw implants^®^) - 3 implants lost	Not mentioned	Not mentioned	Not mentioned	Not mentioned	Shown in clinical photos but not mentioned in treatment	Done in clinical photos but not mentioned in treatment
Deppe et al., 2007 [29]	Group 1 (control): - 19 (17 IMZ^®^, 2 Frialit-2^®^) - 3 implants lost Group 2 (bone augmentation, no laser): 15 (7 IMZ^®^, 5 Frialit-2^®^, 2 Brånemark^®^) - 4 implants lost Group 3 (laser and soft tissue resection): 22 (13 IMZ^®^, 4 Frialit-2^®^, 2 Brånemark^®^, 3 Straumann^®^ screw-type) - 2 implants lost Group 4 (laser and bone augmentation) :17 (11 IMZ^®^, 3 Frialit-2^®^, 2 Brånemark^®^, 1 Straumann^®^ screw-type) - 4 implants lost	Patients with screw-retained prostheses received bone augmentation, implants were submerged with healing time of 4 mos before the implants were reloaded For patients with cemented restorations, soft tissue was resected following decontamination, and implants reloaded immediately after the decontamination	Not mentioned	Screw-retained or cement-retained	Not done	All screw-retained prostheses were removed Cemented prostheses were left in situ	Not done
Nicholson et al., 2014 [30]	Not specified	Not mentioned	3 mos to 16 yrs	Not mentioned	Occlusal adjustment is part of the LAPIP protocol	Not mentioned	Not done
Norton 2017 [31]	27 implants, 2 patients were lost to final follow-up: 1 patient with 2 implants after her 3-mos review, and 1 patient with 1 implant after implant removal due to persistent discomfort at 6 mos	Not mentioned	Not mentioned	Not mentioned	Not done	Documented clinical case showed prosthesis removed, but not mentioned for other cases	Not done
Peng and Tomov 2012 [32]	128 implants: - implant details not specified - no implants were lost	Not mentioned	Not mentioned	Not mentioned	Not done	Suprastructures removed before baseline measurements and before surgical phase	Not done
Renvert et al., 2011 [22]	Air abrasive gp: 45 (29 machined surface, 16 medium rough surface) Laser gp: 55 (41 machined surface, 14 medium rough surface) - no implants were lost	Superstructures replaced and loaded right after treatment	Not mentioned	Not mentioned	Not mentioned	Suprastructures removed before baseline measurements and for treatments Remounted after treatment	Not mentioned
Romanos et al., 2008 [14]	19 implants: 14 Ankylos^®^, 3 ITI^®^, 2 IMZ^®^	4 implants immediately loaded with final restoration after bone graft 12 implants submerged after bone graft	Not mentioned	Not mentioned	Not mentioned	Not mentioned	Not mentioned
Schwarz et al., 2006 [19]	Control: 20 (2 IMZ Twin Plus^®^, 2 ITI SLA TPS^®^, 8 Spline Twist MTX^®^, 4 ZL-Duraplant Ticer^®^, 4 CAMLOG Screw Line^®^) Laser gp: 20 (2 IMZ Twin Plus^®^, 6 ITI SLA TPS^®^, 6 Spline Twist MTX^®^, 4 ZL-Duraplant Ticer^®^, 2 CAMLOG Screw Line^®^)	Not mentioned	Control: 4.2 yrs Laser gp: 5.1 yrs	Not mentioned	Not mentioned	Not mentioned	Not mentioned
Wang et al., 2020 [33]	Control: 12 Laser: 12 Only implants with rough surfaces were included	Not mentioned	At least 6 mos	Not mentioned	Not mentioned	Not done	Supracrestal implantoplasty for peri-implant suprabony defects and infrabony defects debrided with dental scalers or laser prior to bone grafting, bone wax was adapted and fixed in defect to capture the titanium particles

**Table 6:** Radiographic methods and outcomes.

Study	Method of Radiographic Assessment	Radiographic Standardizations	Radiographic Assessment	Radiographic Outcome Compared to Baseline	Radiographic Outcome Compared to Control
Abdul-jabbar et al., 2017 [24]	Mean mesial and distal crestal bone loss (CBL) were recorded in millimeters on digital radiographs using a precalibrated software program (Scion Image, Scion Corp., Fredrick, MD) CBL (peri-implant crestal bone loss): Distance from the widest supracrestal part of the implant to the alveolar crest. Total CBL was determined by averaging the mesial and distal scores of CBL	Standardized digital radiographs using the radiographic paralleling technique and a guiding device at follow-up Calibration of software used was performed using the predefined implant length	Baseline 6 mos	CBL compared to baseline (statistical analysis performed using SPSS v. 18 software, IBM) Control gp 6 mos: Not statistically significant Laser gp 6 mos: Not statistically significant	CBL compared to control Laser gp 6 mos: Not statistically significant Control: Nonsurgical mechanical debridement with plastic curettes
Arısan et al., 2015 [25]	Panoramic radiographs were scanned and visualized using Image J software (NIH, Bethesda, MD) MBL (marginal bone loss): Distance between the implant shoulder and the marginal peri-implant crestal bone was repeated in the distal and mesial of all implants. Measurements were repeated twice, and averaged to yield final values	Measuring tool was calibrated using the known implant length	Baseline 6 mos	MBL compared to baseline (statistical analysis performed using Graphpad Prism 6.0 software, Graphpad Software) Control gp 6 mos: significantly increased Laser gp 6 mos: significantly increased	MBL compared to control Laser gp 6 mos: Significantly increased Control: Conventional scaling and debridement with plastic implant curette
Bach 2009 [26]	Orthopantomograms and dental films assessed visually by clinician	Orthopantomo-grams and dental films in parallel technique, not standardized	Orthopantomo-grams: Baseline Immediate post-op 1 yr Every 2 yrs Dental films: Baseline 4 wks 6 mos 1 yr Every yr	Compared to baseline (no statistical analysis) 1 yr: bone gain in all 17 implants to first thread and implant cervix 5 yrs: bone gain in 12 implants 10 yrs: bone gain in 10 implants >12 yrs: bone gain in 9 implants, horizontal tissue loss in 6 implants at first/second thread In 2 implants a successive loss of the bony supporting tissue led to removal of the artificial abutment in one case after 7 yrs and in another case after 9 yrs	No control
Clem and Gunsolley 2019 [27]	Periapical digital radiographs assessed visually by clinician	Standardized periapical digital radiographs using the Rinn positioner (Dentsply Sirona)	Baseline 3 mos 6 mos 12 mos Every yr	Bone fill compared to baseline (no statistical analysis) 12 mos: positive bone fill in 17 implants (>50% bone fill in 9 implants, 50% bone fill in 3 implants, <50% bone fill in 5 implants, no bone fill in 3 implants, unknown in 3 implants)	No control
Deppe et al., 2005 [28]	Orthopantomograms evaluations for information on the peri-implant marginal bone. Measurements were made with calipers on a back-lit screen in a darkened room. The implant upper edge to the tip of the implant was used as the reference lengthDIB: distance between implant and bone	Standardized orthopantomo-grams, method of standardization not mentioned	Baseline 4 mos 17 mos (6–38 mos)	DIB compared to baseline (statistical analysis performed using Microsoft Excel^®^ version 97) Control gp 4 mos: Improved by 0.4 mm 17 mos: Worsened by 0.3 mm Laser gp 4 mos: Improved by 0.3 mm 17 mos: Improved by 0.4 mm	DIB compared to control Laser gp 4 mos: Not statistically significant 17 mos: Significantly improved Implants lost: Laser gp: 5 Control: 3 Control: conventional decontamination with air-powder abrasive (Prophy-Jet^®^, Denstply)
Deppe et al., 2007 [29]	Radiographic measurements from orthopantomograms of crestal bone level at mesial and distal sites according to Buser et al., [57] Radiographs were not obtained routinely for all patients, since many refused consent DIB: distance from implant shoulder to first bone contact	Standardized orthopantomo-grams taken if consent given, method of standardization not mentioned	Baseline 4 mos 37 mos (5–59 mos)	DIB compared to baseline (statistical analysis performed using MS Excel) Implants in residual bone Laser gp 4 mos: Improved by 0.3 mm 37 mos: Improved by 0.4 mm Control gp 4 mos: Improved by 0.4 mm 37 mos: Worsened by 0.3 mm Implants in augmented bone Laser gp 4 mos: Improved by 4.4 mm 37 mos: Improved by 2.2 mm Control gp 4 mos: Improved by 2.7 mm 37 mos: Improved by 2.1 mm	DIB compared to control Implants in residual bone Laser gp 4 mos: Not significantly different 37 mos: Significantly improved Implants in augmented bone Laser gp 4 mos: Significantly improved 37 mos: Not significantly different Control: conventional decontamination with air-powder abrasive (Prophy-Jet^®^, Dentsply)
Nicholson et al., 2014 [30]	At least two bitewing radiographs, some cases mandibular CT scan or periapical films A technician skilled at reading dental radiographs, identified the baseline alveolar crest and outlined the “areas of changes in radiolucencies” in subsequent images To be more objective a criteria for gray-level to define the boundary of the lesion was identified	Not mentioned	Baseline 2–48 mos	No statistical analysis Rate of recovery range: 0.1–2.4 mm^2^/mon (mean rate: 1.24 mm^2^/mon or 15 mm^2^/yr)Definite trend for larger lesions to heal faster	No control
Norton 2017 [31]	Marginal bone loss on periapical radiographs measured using only contrast, brightness, and sharpness tools in the i-Dixel 3DX software (version 2.2.0.3, Morita)	Periapical radiographs standardized using Rinn device	Baseline 1 yr	Compared to baseline (no statistical analysis) Mesial mean depth reduction: 1.34 mm Distal mean depth reduction: 1.52 mm Mesial defect fill: 27% Distal defect fill: 28%	No control
Peng and Tomov 2012 [32]	Intraoral periapical radiographs analyzed by two calibrated investigators	Intraoral standardized periapical radiographs, holders were used for standardization	Baseline 6 mos	Compared to baseline (no statistical report) Laser gp - Mean bone height loss: 0.1 mm - Proportion with radiographic bone loss (0.1–2.0 mm): 49.3% - Proportion with no radiographic bone change (0.0 mm): 29.3% - Proportion with radiographic bone gain (0.1–3.0 mm): 29.4% Control gp - Mean bone height loss: 0.5 mm - Proportion with radiographic bone loss (0.1–2.0 mm): 74.9% - Proportion with no radiographic bone change (0.0 mm): 4.2% - Proportion with radiographic bone gain (0.1–3.0 mm): 20.9%	Compared to control (no statistical analysis) Laser gp (6 mos): - Less mean bone height loss - Smaller proportion with radiographic bone loss - Larger proportion with no radiographic bone change - Larger proportion with radiographic bone gain Control: Conventional mechanical therapy using ultrasonic device at low settings (PI tip, Piezon^®^ ultrasonic device, EMS) followed by chemical debridement with tetracycline solution
Renvert et al., 2011 [22]	Radiographic digital images assessed using the ImageJ software program 1:43 r (National Institute of Health, Bethesda, MA, USA)	Intraoral standardized radiographs utilizing Eggen holders	Baseline6 mos	Compared to baseline (statistical analysis performed using SPSS PASW software, Statistics 18.0 for MAC, SPSS, Inc.) Laser gp - No differences in alveolar bone changes - Mean bone loss: 0.3 mm - Proportion with radiographic bone loss (0.1–3.0 mm): 58.3% - Proportion with no radiographic bone change (0.0 mm): 2.1% - Proportion with radiographic bone gain (0.1–2.0 mm): 39.6% Control gp - No differences in alveolar bone changes - Mean bone loss: 0.1 mm - Proportion with radiographic bone loss (0.1–3.0 mm): 56.1% - Proportion with no radiographic bone change (0.0 mm): 2.4% - Proportion with radiographic bone gain (0.1–2.0 mm): 41.5%	Compared to control (statistical analysis performed using SPSS PASW software, Statistics 18.0 for MAC, SPSS, Inc.) Data reported not statistically significant Laser gp (6 mos): - More mean bone height loss - Greater proportion with radiographic bone loss - Smaller proportion with no radiographic bone change - Smaller proportion with radiographic bone gain Control: Air abrasive treatment (PERIOFLOW^®^)
Romanos et al., 2008 [14]	Conventional panoramic or periapical radiographs assessed visually by clinician	Not mentioned	Baseline1 mo 3 mos 6 mos 9 mos 12 mos Entire observation period: 27 mos (±17.83 mos)	Compared to baseline Defects with xenogenic bone: Complete bone fill (no statistical analysis) Defects with only autogenous bone graft: At least two-thirds bone fill because of some bone graft resorption (no statistical analysis)	No control
Schwarz et al., 2006 [19]	Periapical radiographs assessed visually by clinician Marginal bone loss as measured from the bone crest to the most coronal bone-to-implant contact	Periapical radiographs were taken using the long-cone parallel technique, standardization not mentioned	Baseline 12 mos	Compared to baseline: No statistical report, no noticeable change in radiographic outcomes	Compared to control: No statistical report, no noticeable change in radiographic outcomes Control: mechanical debridement using plastic curettes followed by pocket irrigation with 0.2% chlorhexidine digluconate solution and 0.2% chlorhexidine gel
Wang et al., 2020 [33]	Linear bone gain in periapical radiographs assessed by determining a constant specific radiographic reference for each patient (platform or porcelain to abutment junction) using MiPACS (Medicor Imaging, Charlotte, North Carolina) Peri-implant defect size measurements were superimposed with 3D Slicer software (Version 4.10.1, Bioinformatics and Computational Biology program, National Institute of Health, USA) and ImageJ software (Version 1.8.0, National Institute of Health, USA).	Standardized radiographs using intraoral periapical digital sensors with customized putty bite blocks for each patient to standardize positioning of the sensor and angle	Baseline 24 wks	Compared to baseline: Radiographic linear bone gain Control: 1.08 mm Laser gp: 1.27 mm Defect size change Laser gp - decreased by 24.46% - more bone gain (no statistical analysis) Control gp - decreased by 15.19% - more bone gain (no statistical analysis)	Compared to control: Radiographic linear bone gain Laser gp: slightly increased, not statistically significant Defect size Laser gp: more bone gain, not statistically significant Control: Open flap mechanical debridement, supracrestal implantoplasty, bone grafting, and acellular dermal matrix membrane without laser therapy

**Table 7:** Other clinical parameters and outcomes.

Study	Bleeding on Probing Compared to Baseline	Bleeding on Probing Compared to Control	Clinical Attachment Level Gain Compared to Baseline	Clinical Attachment Level Gain Compared to Control	Probing Depth Compared to Baseline	Probing Depth Compared to Control	Microbial Analysis Compared to Baseline	Microbial Analysis Compared to Control	Adverse Reactions
Abdul-jabbar et al., 2017 [24]	No statistical analysis Control gp 3 mos: Decreased 32.1% 6 mos: Decreased 39.8% Laser gp 3 mos: Decreased 44.8% 6 mos: Decreased 39.8% Comparative suppuration results not reported	Statistical analysis performed using SPSS v.18 software, IBM Baseline: No significant difference 3 mos: Signifi-cantly lower 6 mos: No significant difference Compara-tive suppura-tion results not reported	Not assessed	Not assessed	No statistical analysis Control gp 3 mos: Decreased 1.1 mm 6 mos: Decreased 1.6 mm Laser gp 3 mos: Decreased 2.9 mm 6 mos: Decreased 2.8 mm	Statistical analysis performed using SPSS v.18 software, IBM Baseline: No significant difference 3 mos: Significantly lower 6 mos: No significant difference	Not done	Not done	Not mentioned
Arısan et al., 2015 [25]	Statistical analysis performed with Graphpad Prism 6.0 software, Graphpad Software Control gp 1 mo: Significantly decreased 6 mos: No significant difference Laser gp 1 mo: Significantly decreased 6 mos: No significant difference	Statistical analysis performed with Graphpad Prism 6.0 software, Graphpad Software No significant difference	Not assessed	Not assessed	Statistical analysis performed with Graphpad Prism 6.0 software, Graphpad Software Control gp 1 mo: Significantly decreased 6 mos: Significantly increased Laser gp 1 mo: Significantly decreased 6 mos: Significantly increased	Statistical analysis performed with Graphpad Prism 6.0 software, Graphpad Software Baseline: No significant difference 1 mon: No significant difference 6 mos: No significant difference	Statistical analysis performed with Graphpad Prism 6.0 software, Graphpad Software Control gp 1 mo: No significant difference Laser gp 1 mo: No significant difference	Statistical analysis performed with Graphpad Prism 6.0 software, Graphpad Software Not statistically significant	No complications or negative outcomes
Bach 2009 [26]	Not assessed	No control	Not mentioned	No control	Not assessed	No control	*P. gingivalis* almost completely eliminated during the whole examination period, and a significant reduction of other anaerobe, gram-negative bacteria	No control	Not mentioned
Clem and Gunsolley 2019 [27]	Not reported	No control	Not assessed	No control	Statistical analysis with Tukey *t*-test and ANOVA For probings <6 mm 6 mos: No statistically significant improvement 12 mos: No statistically significant improvement For probings ≥ 6 mm 6 mos: Statistically significant improvement 12 mos: Statistically significant improvement	No control	Not done	No control	Not mentioned
Deppe et al., 2005 [28] Data before surgical interven-tion was used as the baseline in this table	No statistical analysis Sulcus Bleeding index (SBI): Control gp 4 mos: Increased 17 mos: Increased Laser gp 4 mos: Increased 17 mos: Increased	No statistical analysis 4 mos: Decreased 17 mos: Increased	Statistical analysis performed with Microsoft Excel version 97 software Control gp 4 mos: Improved 17 mos: Improved Laser gp 4 mos: Improved 17 mos: Improved	Statistical analysis performed with Microsoft Excel version 97 software 4 mos: Significantly better attachment levels 17 mos: No significant difference	No statistical analysis Control gp 4 mos: Decreased 17 mos: Decreased Laser gp 4 mos: Decreased 17 mos: Decreased	No statistical analysis 4 mos: Decreased 17 mos: Decreased	Not done	Not done	No adverse effects
Deppe et al., 2007 [29] Data before surgical interven-tion was used as the baseline in this table	No statistical analysis Sulcus Bleeding index (SBI): Implants in residual bone Control gp 4 mos: Increased 37 mos: Increased Laser gp 4 mos: Increased 37 mos: Increased Implants in augmented bone Control gp 4 mos: Increased 37 mos: Increased Laser gp 4 mos: Decreased 37 mos: Increased	No statistical analysis Sulcus Bleeding index (SBI): Implants in residual bone 4 mos: Decreased 37 mos: Increased Implants in augmen-ted bone 4 mos: Increased 37 mos: Decreased	No statistical analysis Implants in residual bone Control gp 4 mos: Decreased 37 mos: No change Laser gp 4 mos: Decreased 37 mos: Decreased Implants in augmented bone Control gp 4 mos: Decreased 37 mos: Decreased Laser gp 4 mos: Decreased 37 mos: Decreased	Statistical analysis performed with MS Excel software Implants in residual bone 4 mos: Significantly improved 37 mos: Significantly improved Implants in augmented bone 4 mos: Significantly improved 37 mos: No significant difference	No statistical analysis Implants in residual bone Control gp 4 mos: Decreased 37 mos: Decreased Laser gp 4 mos: Decreased 37 mos: Decreased Implants in augmented bone Control gp 4 mos: Decreased 37 mos: Decreased Laser gp 4 mos: Decreased 37 mos: Decreased	No statistical analysis Implants in residual bone 4 mos: Decreased 17 mos: Decreased Implants in augmented bone 4 mos: Decreased 17 mos: No difference	Not done	Not done	Typical postoperative edema 1 patient in conventional augmented group developed severe infection, resulting in total loss of augmentation and all 4 implants within the first weeks after surgery In 1 patient in laser augmented treatment, most augmentation and all 4 implants were lost about 10 months after treatment because of a chronic infection
Nicholson et al., 2014 [30]	Not mentioned	No control	Not mentioned	No control	Not reported	No control	Not done	Not done	Not mentioned
Norton 2017 [31]	No statistical analysis 1 yr: BOP: 54% reduction Spontaneous bleeding: 80% reduction Spontaneous suppuration: 50% reduction	No control	Not assessed	No control	No statistical analysis 1 yr: Reduced 2.8 mm	No control	Not done	Not done	Not mentioned
Peng and Tomov 2012 [32]	Statistical analysis performed with SPSS software Control gp 6 mos: Significantly reduced Laser gp 6 mos: Significantly reduced	6 mos: Significantly reduced	Not assessed	Not assessed	No statistical analysis Control gp 6 mos: Reduced 0.8 mm Laser gp 6 mos: Reduced 1.7 mm	Not mentioned	Not assessed	Not assessed	Not mentioned
Renvert et al., 2011 [22]	Statistical analysis performed with SPSS PASW Statistics 18.0 for MAC software, SPSS Inc. BOP: Implant level Control gp 6 mos: Significantly decreased Laser gp 6 mos: Significantly decreased Suppuration: Control gp 6 mos: Significantly decreased Laser gp 6 mos: Significantly decreased	Statistical analysis performed with SPSS PASW Statistics 18.0 for MAC software, SPSS Inc. Implant level BOP: Not Statisti-cally significant Subject level BOP: Not statisti-cally significant Suppura-tion: Not statisti-cally significant	Not mentioned	Not mentioned	No statistical analysis Implant level Control gp 6 mos: Reduced 0.9 mm Laser gp 6 mos: Reduced 0.8 mm Subject level Control gp 6 mos ≥ 1 mm reduction: 38% Laser gp 6 mos ≥ 1 mm reduction: 25%	Statistical analysis performed with SPSS PASW Statistics 18.0 for MAC software, SPSS Inc. Not statistically significant	Not mentioned	Not mentioned	No serious adverse events
Romanos et al., 2008 [14]	Statistical analysis performed but methodology not described Sulcus bleeding index (SBI): Significantly reduced	No control	Not mentioned	No control	Statistical analysis performed but methodology not described Significantly reduced	No control	Not mentioned	Not mentioned	No peri-implant bleeding or suppuration
Schwarz et al., 2006 [19]	Statistical analysis performed with SPSS 14.0 software, SPSS Mean BOP Control gp 3 mos: Significantly reduced 6 mos: Significantly reduced 12 mos: Significantly reduced Laser gp 3 mos: Significantly reduced 6 mos: Significantly reduced 12 mos: Significantly reduced	Statistical analysis performed with SPSS 14.0 software, SPSS 3 mos: Signifi-cantly reduced 6 mos: Signifi-cantly reduced 12 mos: Not mentioned	Statistical analysis performed with SPSS 14.0 software, SPSS Control gp 3 mos: Significant gain 6 mos: Significant gain 12 mos: Not significant Laser gp 3 mos: Significant gain 6 mos: Significant gain 12 mos: Not significant	Statistical analysis performed with SPSS 14.0 software, SPSS 3 mos: Not significant 6 mos: Not significant 12 mos: Not significant	Statistical analysis performed with SPSS 14.0 software, SPSS Control gp 3 mos: Significantly reduced 6 mos: Significantly reduced 12 mos: Significantly reduced Laser gp 3 mos: Significantly reduced 6 mos: Significantly reduced 12 mos: Significantly reduced	Statistical analysis performed with SPSS 14.0 software, SPSS 3 mos: Not significant 6 mos: Not significant 12 mos: Not significant	Not mentioned	Not mentioned	Generally uneventful Pus formation in 2 control patients Laser perforation of buccal keratinized mucosa and gingival recession in 1 laser patient At 12 mos, both groups were discontinued from the study due to increasing BOP and a slight loss of mean CAL. These patients received further periimplantitis laser treatment and subsequent bone augmentation
Wang et al., 2020 [33]	Statistical analysis performed with SPSS 20 software (IBM, USA) BOP &GI: Control gp 24 wks: Significantly reduced Laser gp 24 wks: Significantly reduced	Statistical analysis performed with SPSS 20 software (IBM, USA) Laser gp 24 wks: Not significant	Statistical analysis performed with SPSS 20 software (IBM, USA) Control gp 24 wks: Significant increase Laser gp 24 wks: Significant increase	Statistical analysis performed with SPSS 20 software (IBM, USA) Laser gp 24 wks: Increase, not significant	Statistical analysis performed with SPSS 20 software (IBM, USA) Control gp 24 wks: Significantly reduced Laser gp 24 wks: Significantly reduced	Statistical analyses performed using SPSS 20 (IBM, USA). Laser gp 24 wks: Significantly reduced	Not mentioned	Not mentioned	Membrane exposure significantly reduced the PD reduction and CAL gain, this was clinically significant

**Table 8:** Clinical significance of laser therapy ≥ 6 months follow-up.

Study	Type of Laser	Inflamma-tion (BOP/SBI and/or Suppuration) Compared to Baseline	Inflamma-tion (BOP/SBI and/or Suppuration) Compared to Control	Probing Depth Compared to Baseline	Probing Depth Compared to Control	Bony Defect Compared to Baseline	Bony Defect Compared to Control	Control
Abdul-jabbar et al., 2017 [24]	Nd:YAG (at 6 mos)	Reduced, significance not analyzed	Not Significant	Reduced, significance not analyzed	Not Significant	Not Significant bone loss	Not Significant bone loss	Mechanical debridement with plastic curettes
Arısan et al., 2015 [25]	810-nm Diode (at 6 mos)	Not Significant	Not Significant	Significant reduction	Not Significant	Significant bone loss	Significant bone loss	Mechanical debridement with plastic curettes
Bach 2009 [26]	810-nm Diode (at 12 mos)	Not reported	No control	Not reported	No control	Bone gain, significance not analyzed	No control	No control
Clem and Gunsolley 2019 [27]	Er:YAG (at 12 mos)	Not reported	No control	Probings < 6 mm: Not Significant	No control	Bone gain, significance not analyzed	No control	No control

				Probings ≥ 6 mm: Significant reduction				
Deppe et al., 2005 [28] Data before surgical interven-tion was used as the baseline in this table	10,600-nm CO_2_ (at mean 17 mos)	Increased, significance not analyzed	Increased, significance not analyzed	Reduced, significance not analyzed	Reduced, significance not analyzed	Bone gain, significance not analyzed	Significant bone gain	Air-powder abrasive
Deppe et al., 2007 [29] Data before surgical interven-tion was used as the baseline in this table	10,600-nm CO_2_ (at mean 37 mos)	Increased, significance not analyzed	Tissue resection gp: Increased, significance not analyzed Augmented bone gp: Decreased, significance not analyzed	Reduced, significance not analyzed	Tissue resection gp: Reduced, significance not analyzed Augmented bone gp: No change, significance not analyzed	Bone gain, significance not analyzed		Air-powder abrasive
							Tissue resection gp: Significant bone gain	

							Augmented bone gp: No significant bone gain	

Nicholson et al., 2014 [30]	Nd:YAG (at 2 to 48 mos)	Not reported	No control	Not reported	No control	Bone gain, significance not analyzed	No control	No control
Norton 2017 [31]	Er:YAG (at 12 mos)	Reduced, significance not analyzed	No control	Reduced, significance not analyzed	No control	Bone gain, significance not analyzed	No control	No control
Peng and Tomov 2012 [32]	Er:YAG (at 6 mos)	Significant reduction	Significant reduction	Reduced, significance not analyzed	Reduced, significance not analyzed	Bone loss, significance not analyzed	Less bone loss, significance not analyzed	Mechanical therapy with ultrasonics followed by chemical debridement
Renvert et al., 2011 [22]	Er:YAG (at 6 mos)	Significant reduction	Not Significant	Reduced, significance not analyzed	Not Significant	Not Significant bone loss	Not significant bone loss	Air abrasive treatment
Romanos et al., 2008 [14]	10,600-nm CO_2_ (at mean 27 mos)	Significant reduction	No control	Significant reduction	No control	Bone gain, significance not analyzed	No control	No control
Schwarz et al., 2006 [19]	Er:YAG (at 6 and 12 mos)	Significant reduction	Significant reduction (not reported at 12 mos)	Significant reduction	Not Significant	Not Significant (NR) (at 12 mos)	Not Significant (NR) (at 12 mos)	Mechanical debridement with plastic curettes and chlorhexidine pocket irrigation
Wang et al., 2020 [33]	Er:YAG (at 24 wks)	Significant reduction	Reduced, not significant	Significant reduction	Significant reduction	Bone gain, significance not analyzed	Not significant bone gain	Same as test group, but no laser therapy
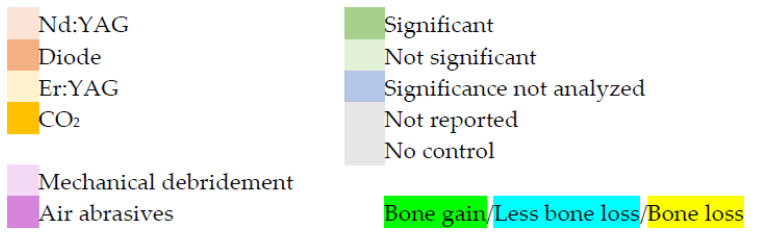								

**Table 9:** Recommendations for future studies.

Range of Variables That May Apply to Laser-Based Studies of Peri-Implantitis Treatment
Study Design
Start and end dates (and/or duration) specified Experimental and control groups adequately described Inclusion and exclusion criteria specified Antibiotics and/or oral irrigants specified Biomaterials, bone grafts, regenerative therapies specified Follow-up care and time intervals described Home care instructions described Flap closure methods described Statistical methods and software detailed Number and locations of probing per implant Type of peri-implant bone defect described
Investigators
Adequately calibrated in research design, diagnosis, clinical diagnosis, treatment methods Level of experience with treatment methods
Patients
Inclusion and exclusion criteria Sample size of control and treatment groups Gender Age range and mean Health status Dropouts explained
Clinical Parameters
Gingival index Gingival bleeding index Probing depth Clinical attachment level Mobility Bleeding on probing or sulcular bleeding index Suppuration Plaque index Gingival recession Microbial analysis
Radiographic Analysis
Radiograph type and method Radiographic standardization method described Software used Analyses defined (e.g., crestal bone loss, marginal bone loss) Time intervals (e.g., baseline, 6 months, 1 year, additional years) Statistical analysis described Trends in healing
Implant
Manufacturer Number and locations Shape (e.g., two-piece, tapered root form) Type (e.g., platform-switched, machined surface, rough surface, enhanced surface) Duration of implant function prior to treatment (range, mean) Loading protocol at initial placement or after treatment Restoration connection (cement, screw) retention
Risk of Bias Assessment
Selection Bias: Adequately randomized Allocation adequately concealed Comparison groups are appropriate Confounding Bias: Confounding or modifying variables accounted for Performance Bias: Adequately blinded Attrition/Exclusion Bias: Outcome data complete Detection Bias: Exposure characterization confidence—Treatment consistently administered Outcome assessment confidence—Outcomes assessed using well-established methods Selective Reporting Bias: All measured outcomes reported and statistically analyzed Statistical significance specified for all measured outcomes Outcomes, both short-term and long-term Complications (if any) and management thereof Adverse and unanticipated events (if any) and management thereof Other Bias: Statistical methods appropriate Study protocol adhered to Conflicts of interest and/or dual commitments disclosed Commercial support disclosed
Laser Device Information
Manufacturer Model Beam delivery system (e.g., articulating arm, waveguide, optical fiber)
Laser Irradiation Parameters
Center wavelength (nm) Spectral bandwidth (nm) Operating mode (e.g., continuous wave (CW), pulsed) Pulse frequency (Hz) Pulse duration (µsec) Duty cycle (%) Peak radiant power (W) Average radiant power (W) Beam profile (e.g., Gaussian, Top Hat) Water cooling setting during treatment Air cooling setting during treatment
Laser Treatment Parameters
Rationale for the chosen parameters and dosage Beam focused or unfocused Beam shape and/or diameter (spot size) at target area (cm^2^) Irradiance at target (mW/cm^2^) Exposure duration (sec) Radiant exposure (J/cm^2^) Radiant energy (J) Number of points irradiated Area irradiated (cm^2^) Application technique (contact, noncontact with working distance) Angle of beam or tip Movement and motion of beam or tip Tip composition and description Tip initiation Number and frequency of treatment sessions Intervals between treatments Total radiant energy (J)
Method of Laser Use during Peri-Implantitis Treatment
Treatment prior to laser irradiation, if any Target (e.g., tissue only, implant only, both) End point specified (e.g., timed exposure duration, number of passes, change in implant surface characteristics) Adjunctive treatment prior to, during, or after laser irradiation (e.g., preprocedural rinse, flap reflection, mechanical debridement (hand and/or ultrasonic instrumentation) described, air abrasive treatment, antimicrobial rinse, implantoplasty, occlusal adjustment, photobiomodulation, photodynamic therapy, bone decortication) Hand instrumentation described (e.g., plastic or titanium curettes) Ultrasonic instrumentation described (device, tips, irrigant) Air abrasive instrumentation described (device, powder, flow rate) Granulation and granulomatous tissue removed or retained Suprastructure or superstructure removed during treatment Clot formation Laser parameters varied according to specific application Biomaterials

## Data Availability

The data supporting the findings of this systematic review are available within the article.
